# P-544. Respiratory and Nonrespiratory Complications in Children Hospitalized with Influenza in the Post-2009 H1N1 Pandemic Era

**DOI:** 10.1093/ofid/ofaf695.759

**Published:** 2026-01-11

**Authors:** Satoshi Kamidani, Lucy S Witt, Annabel Patterson, Emma G Turner, Emily Fawcett, Shua J Chai, Isaac Armistead, James Meek, Lauren Leegwater, Melissa McMahon, Marc Martinez, Jemma Rowlands, Melissa Sutton, Isabella Reyes, Camden Castagna-McLeod, Alissa O’Halloran, Catherine Bozio, Kyle P P Openo

**Affiliations:** Emory University School of Medicine and Children's Healthcare of Atlanta, Suwanee, GA; Emory University, Atlanta, Georgia; Emory University, Atlanta, Georgia; Emory University, Atlanta, Georgia; Emory University School of Medicine, Atlanta, Georgia; California Emerging Infections Program, Oakland, CA; Division of State and Local Readiness, CDC, Atlanta, Atlanta, Georgia; Colorado Department of Public Health and Environment, Denver, Colorado; Connecticut Emerging Infections Program, Yale School of Public Health, New Haven, Connecticut; Michigan Department of Health & Human Services, Grand Rapids, Michigan; Minnesota Department of Health, St. Paul, Minnesota; NMDOH, Albuquerque, New Mexico; New York State Department of Health, Albany, New York; Public Health Division, Oregon Health Authority, Portland, Oregon; Salt Lake County Health Department, Sale Lake City, Utah; Oak Ridge Institute for Science and Education, Oak Ridge, Tennessee, USA and Influenza Division, National Center for Immunization and Respiratory Diseases, Centers for Disease Control and Prevention, Atlanta, Georgia, USA, Atlanta, Georgia; CDC, Atlanta, GA; CDC, Atlanta, GA; Emory University School of Medicine, Atlanta, Georgia

## Abstract

**Background:**

Despite the known spectrum of influenza-associated complications, limited epidemiologic data describe the frequency and outcomes of respiratory and nonrespiratory complications in pediatric patients, particularly in the post-2009 H1N1 pandemic era. Analyzing recent data on hospitalized children may offer important insights into the full spectrum of influenza-related complications.

**Figure 1.**
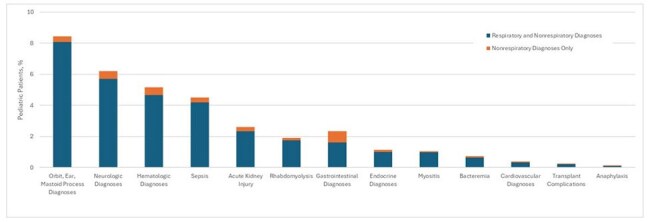
Nonrespiratory Diagnoses Among Children <18 Years Hospitalized with Laboratory-Confirmed Influenza in the Influenza Hospitalization Surveillance Network (FluSurv-NET), United States, 2010-2023 seasons.

**Table 1.**
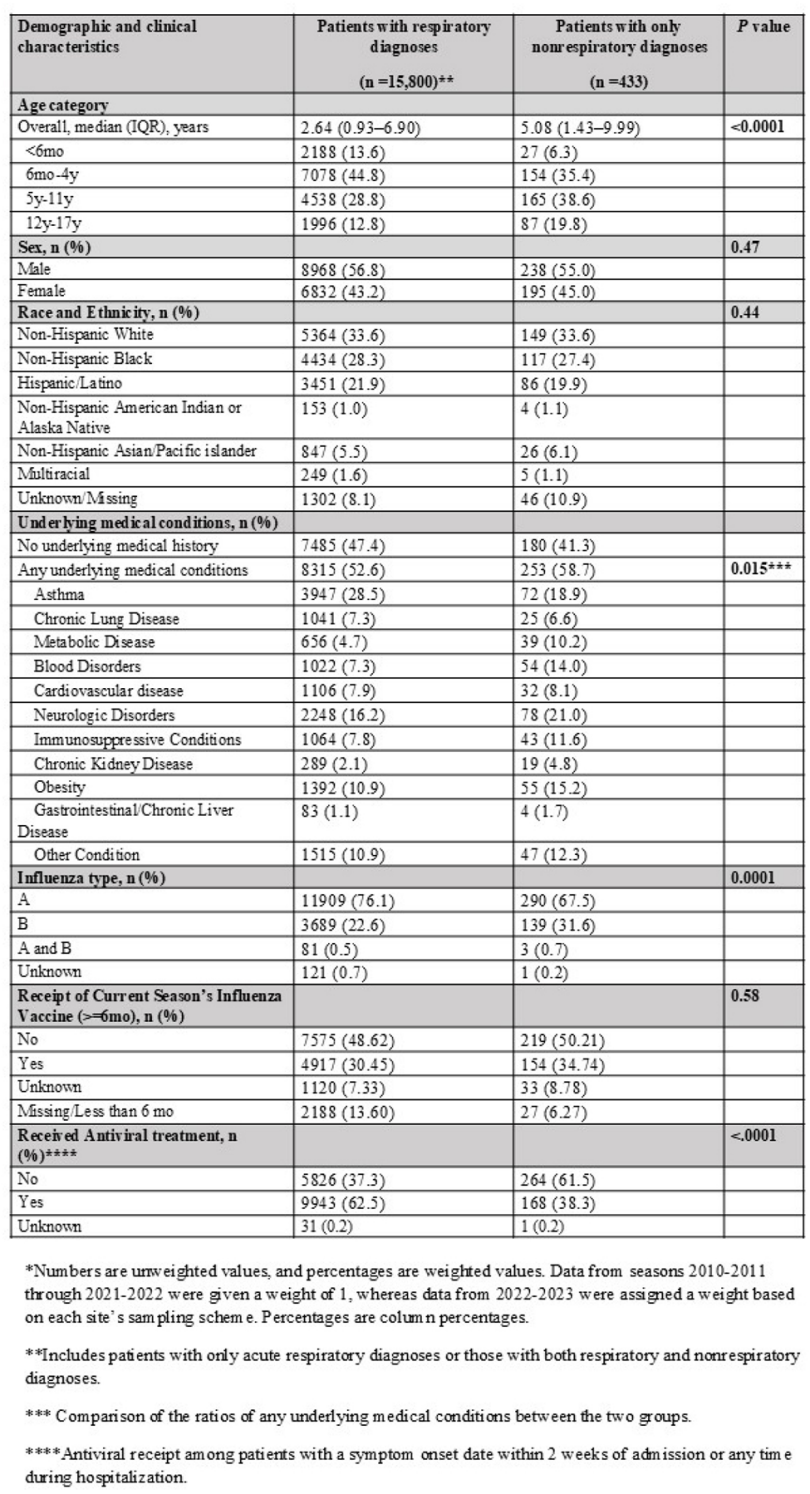
Characteristics of Patients with Acute Respiratory Diagnoses or with Nonrespiratory Diagnoses Among Hospitalized Children with Influenza within FluSurv-NET, United States, 2010-2023.*

**Methods:**

Children aged < 18 years hospitalized with laboratory-confirmed influenza during the 2010–2011 through 2022–2023 influenza seasons were identified through the Influenza Hospitalization Surveillance Network (FluSurv-NET), which covers ∼9% of the U.S. population. Acute respiratory and nonrespiratory discharge diagnoses were classified based on the International Classification of Diseases, 9th and 10th Revisions (ICD-9/10). Descriptive and bivariate analyses were conducted on demographics, underlying conditions, and in-hospital outcomes by diagnosis type.

**Table 2.**
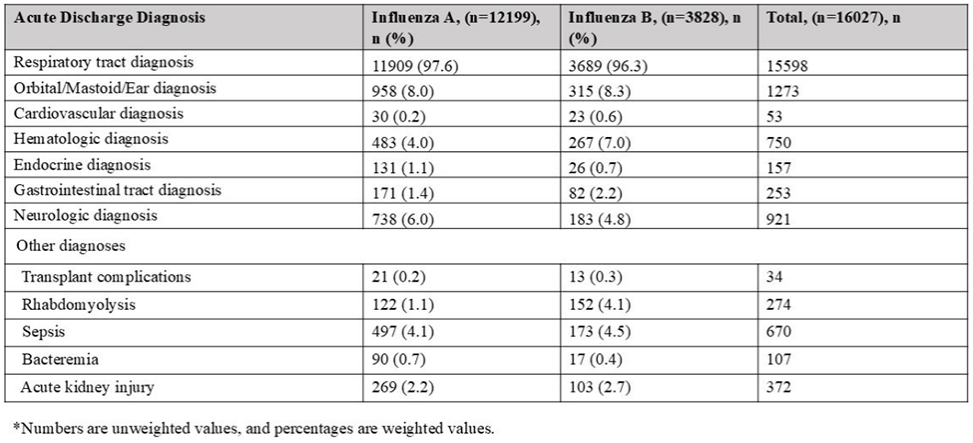
Hospitalizations with Acute Diagnoses by Influenza Type in FluSurv-NET, United States, 2010-2023 seasons.*

**Table 3.**
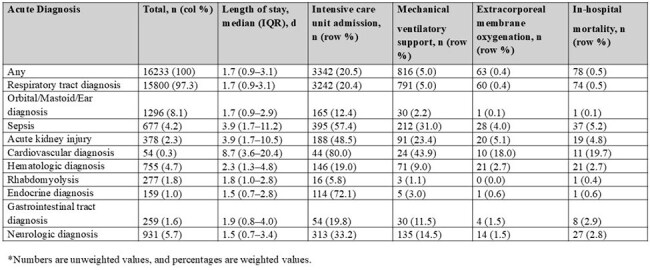
In-Hospital Outcomes Among Children Hospitalized with Laboratory-Confirmed Influenza by Acute Diagnosis Category in FluSurv-NET, United States, 2010-2023 seasons.*

**Results:**

Among 17442 hospitalized children, 16233 had ≥1 ICD-coded acute diagnosis and were included. Of these, 15800 (97%) had a respiratory diagnosis and 4469 (28%) had a nonrespiratory diagnosis, including 433 (2.7%) with only nonrespiratory diagnoses. The most common nonrespiratory diagnosis categories included orbit/ear/mastoid process disorders (8.4%), neurologic (6.2%), hematologic (5.2%), and sepsis (4.5%) (Fig.1). Compared to children with respiratory diagnoses, those with only nonrespiratory diagnoses were significantly older (median 5.1 vs. 2.6 years; P < .0001), more frequently infected with influenza B virus (32% vs. 23%; P < .0001), and more likely to have underlying medical conditions (59% vs. 53%; P =.015) (Tables 1 and 2). Antiviral use was less common in the nonrespiratory group (38% vs. 63%; P< .001). Among influenza-associated acute diagnoses, children with sepsis, cardiovascular disease, and acute kidney injury had high frequencies of severe outcomes (Table 3).

**Conclusion:**

This population-based study highlights the clinical characteristics and burden of respiratory and nonrespiratory complications among children hospitalized with influenza. Enhanced awareness and timely recognition of these complications may improve clinical management and promote antiviral use.

**Disclosures:**

Satoshi Kamidani, MD, PhD, Bavarian Nordic: Grant/Research Support|Meissa: Grant/Research Support|Moderna: Grant/Research Support|Pfizer: Grant/Research Support|Sanofi: Grant/Research Support Lucy S. Witt, MD, MPH, Merck & Co: Grant/Research Support Melissa Sutton, MD, MPH, Centers for Disease Control and Prevention Emerging Infections Program: Grant/Research Support

